# The Crimean-Congo haemorrhagic fever virus hijacks the liver lipid metabolic pathway for virion production

**DOI:** 10.1080/22221751.2026.2645855

**Published:** 2026-03-30

**Authors:** Anupriya Gautam, Li Zhong, Eva Ogire, Sergueï Bodoirat, Ilary Riedmiller, Willem J. Sander, Bertrand Boson, Apoorv Gandhi, Julien Burlaud-Gaillard, Fouzia Amirache, Vincent Legros, Philippe Roingeard, Vincent Lotteau, Cyrille Mathieu, François-Loïc Cosset, Solène Denolly

**Affiliations:** a CIRI – Centre International de Recherche en Infectiologie, Université de Lyon, Université Claude Bernard Lyon 1, Inserm, U1111, CNRS, UMR5308, ENS de Lyon, Lyon, France; b Morphogénèse et Antigénicité du VIH et des Virus des Hépatites (MAVIVH), Université de Tours and CHRU de Tours, Tours, France; c UMS Inserm 61 ASB, Université et CHU de Tours, Tours, France; d Campus vétérinaire de Lyon, VetAgro Sup, Université de Lyon, Lyon, France; e Laboratoire P4-Jean Mérieux, Inserm, Lyon, France; f Centre de Recherche en Cancérologie de Lyon, INSERM U1052-CNRS UMR5286, Université de Lyon, Centre Léon Bérard, Lyon, France

**Keywords:** CCHFV, lipoprotein, hepatocyte, ApoB, DGAT-1, MTP, antiviral

## Abstract

The Crimean-Congo Haemorrhagic Fever Virus (CCHFV), a tri-segmented negative-strand virus that belongs to the *Orthonairovirus* genus, is highly pathogenic in humans but not in other host species. Following infection in humans, CCHFV disseminates widely but replicates most strongly in hepatocytes, indicating a preferential liver tropism. As hepatocytes are the primary site for production and secretion of lipids, here we sought to characterize the interplay between CCHFV and lipoprotein metabolism in hepatocytes. First, we found that CCHFV particles display a heterogeneous profile of density, suggesting various virion compositions. Next, we showed that several lipoprotein components are associated with viral particles. Additionally, we found that pharmacological inhibition or down-regulation of the host factors involved in lipoprotein biogenesis and lipid metabolism could impair CCHFV infection. Our results, therefore, by revealing a close interplay between liver lipid metabolism and CCHFV, highlight the potential of repurposing existing lipid-modulating drugs and allow designing new interventions to curb CCHFV infections.

## Introduction

Crimean-Congo haemorrhagic fever virus (CCHFV) is one of the most widespread tick-borne viruses affecting humans, causing severe outbreaks with high case fatality rates ranging from 10% to 40% [1]. The virus is primarily maintained in nature through a cycle involving *Hyalomma* ticks and various vertebrate hosts, including cattle, sheep, goats, wild mammals, and migrating birds. Migratory birds, by transporting infected ticks from Africa to Europe, and other vertebrates can act as amplifying reservoirs [2]. Human infection typically occurs through tick bites or through contact with infected animal blood or tissues, resulting in symptoms that range from mild febrile illness to severe haemorrhagic manifestations and multi-organ failure [3]. Following infection, the virus disseminates widely but replicates most strongly in hepatocytes, indicating a preferential liver tropism [4]. CCHFV is endemic across Africa, Eastern and South-Western Europe, the Middle East, and parts of Asia, with its geographic range expanding due to climate change, animal movement, and global trade [5,6]. Despite its significant public health threat, there is currently neither an approved vaccine nor a specific antiviral therapy for CCHFV, making it essential to deepen our understanding of its biology [3].

CCHFV is an enveloped, negative-sense RNA virus of the family Nairoviridae within the order Hareavirales (class Bunyaviricetes). It uses the low-density lipoprotein receptor (LDL-R) to initiate infection, as shown by us and others [7–9]. Its tripartite genome encodes the structural proteins nucleoprotein (NP), glycoproteins (Gn and Gc), and the RNA-dependent RNA polymerase (L). The structure of the CCHFV surface glycoproteins complex has been proposed [10]; however, the organization of the virion surface remains difficult to understand. Indeed, there is currently no clear model of the surface topology of the CCHF virus particle, since, in addition to Gn and Gc, which are classically found on *Bunyaviricetes* members, CCHFV displays on its surface a host factor, the apolipoprotein E [7]. Apolipoproteins are a class of amphipathic proteins that bind lipids to form lipoproteins, playing a fundamental role in lipid transport, metabolism, and cellular signalling. These proteins, such as apolipoprotein E (apoE) and apolipoprotein B (apoB), function as structural and/or regulatory components of lipoprotein particles and act as ligands for LDL-R-mediated endocytosis, regulating cholesterol homeostasis and inflammatory responses [11].

Although hepatocytes readily yield infectious CCHFV particles and are the primary site for the assembly and secretion of lipoproteins, the role of lipoprotein-related pathways in CCHFV production and infection remains unexplored, highlighting an important gap in our understanding of its pathogenesis. As CCHFV produced from non-hepatic cells is less dependent on LDL-R for cell entry [7], we surmise that hepatocytes may provide an “imprinting” to optimize the infectivity of produced viral particles, at either the assembly, secretion, or maturation steps in a manner linked to lipoprotein metabolism. Thus, should this hypothesis be true, CCHFV production could be subjected to association or recruitment with different types of lipoprotein components during either production or maturation of viral particles in infected hepatocytes.

Here, we addressed the potential connection between CCHFV and the lipoprotein production pathway. CCHFV is a pathogen classified as RG4 (risk group) and must be handled under maximum containment conditions, i.e. in a BSL-4 (Biosafety Level-4) laboratory, which is a rare and very expensive resource. Thus, for parts of this study, we employed CCHFV minigenome particles (CCHFVmg), corresponding to previously described CCHFV transcription and entry competent virus-like particles [12], which can be handled under BSL-2 conditions and recapitulate authentic virus structures [13], allowing the study of all steps of the viral life cycle [7,14]. Importantly, we validated our key findings using *bona fide* wild-type CCHFV under BSL-4 containment to ensure that observations from the CCHFVmg system reflect authentic viral behaviour. Our report demonstrates that CCHFV exploits major factors of lipoprotein biogenesis and lipid metabolism for the assembly and post-egress modifications of its particles, which allowed us to identify an array of inhibitors against these host factors that potently inhibited the production of infectious CCHFV particles.

## Materials and methods

### Cell culture and reagents

Huh-7.5 hepato-carcinoma (kind gift from C. Rice) was grown in Dulbecco’s modified Eagle’s medium (DMEM) complemented with 10% foetal bovine serum (FBS), and 1% penicillin–streptomycin. All the cells were grown in a 37°C incubator with 5% CO_2_.

For preparation of human serum (HS), blood from healthy donors retrieved from the national blood collection was incubated on ice for 2 h and centrifuged at 4,000 rpm (revolutions per minute) for 20 min, and the supernatants were harvested and stored at –80°C. The HS used in this study consisted of pools of four specimens from different donors.

Three different batches of PHH from healthy donors (BD Biosciences and TRL-Biopredic) were thawed in OneStep PHep Thawing Medium (Biopredic) and centrifuged at 100 xg for 10 min at 20°C. Cells were then counted and seeded overnight in collagen-coated plates in PHH medium corresponding to William’s E medium (Gibco) supplemented with 10% FBS, 1 µg/mL BSA, 5 µg/mL bovine insulin, 1 × 10^−6^ M Dexamethasone (Sigma Aldrich), 1 × 10^−8^ M 3.3 triiodo-L-thyronine, 5 µg/mL apotransferrin, 1% of non-essential amino acids (Gibco), 1% of Glutamine (Gibco), and 1% Penicillin–Streptomycin solution (Gibco). 16 h later, PHH were washed and cultured in PHH medium.

### Plasmids and constructs

The constructs encoding wild-type CCHFV strain IbAr10200 L polymerase (pCAGGS-V5-L), CCHFV nucleoprotein NP (pCAGGS-NP), CCHFV-specific eGFP-expressing minigenome (pT7RiboSM2_vL_eGFP), nanoluciferase (nanoLuc)-expressing minigenome flanked by L NCR under the control of a T7 promoter (pSMART-LCK_L-Luc), T7 RNA polymerase (pCAGGS-T7), CCHFV M-segment polyprotein (pCAGGS-GPC), and an empty vector without viral genes (pCAGGS) were described previously [12,15] and used for the production of CCHFVmg particles (all kind gifts from Friedemann Weber and Eric Bergeron). pMK-RQ-HAZV resQ S EGFP P2A, pMK-RQ-HAZV M, and pMK-RQ-HAZV L (kind gift from John N. Barr) were used for the production of rescued Hazara virus (HAZV) rHAZV-eGFP [16].

### Antibodies and inhibitors

The list of all antibodies used in this study is available in the Supplementary Table 1.

### Production of CCHFVmg particles

Huh-7.5 cells were seeded in 10 cm dishes and transfected with 3.6 µg of pCAGGS-V5-L, 1.2 µg of pCAGGS-NP, 1.2 µg of pT7riboSM2-vL-GFP or pSMART-LCK_L-Luc, 3 µg of pCAGGS-GPC, 3 µg of pCAGGS-T7 by using TranIT-LT1 transfection reagent (MIR2300, Mirus Bio), and were used in a 1:2 ratio. The transfection media was replaced after 6 h post-transfection with OptiMEM for serum-free conditions, or with OptiMEM supplemented with 10% HS when indicated. Cell supernatants were harvested 72 h post-transfection and filtered through a 0.45 μm filter.

For infection assays with CCHFVmg particles, target cells were pre-transfected using 3.6 µg of pCAGGS-V5-L and 1.2 µg of pCAGGS-NP using TranIT LT1 transfection reagent. 6 h post-transfection, cells were seeded in 24, 48, or 96-well plates in OptiMEM. 24 h post-transfection, cells were transduced with serial dilutions of particles, corresponding to MOIs of 2–0.02 (for CCHFVmg-GFP particles) or to relative light units (RLU)-per-cell of 500–1. For each dilution, technical replicates were performed. 24 h post transduction, transduced cells were fixed, and the percentage of GFP-positive cells was assessed by flow cytometry (MACSQuant® VYB Flow Cytometer; Miltenyi Biotec). Data were analysed with FlowJo software (BD Biosciences). The viral titre was determined after selection of dilutions within the linear range of percentages of positive cells.

For incubation experiments, CCHFVmg particles were produced in OptiMEM and then incubated with OptiMEM vs. HS (10%) at 37 °C for 6 h.

The raw infectious titres of CCHFVmg particles are provided in the Supplemental Figure 1.

**Figure 1.**
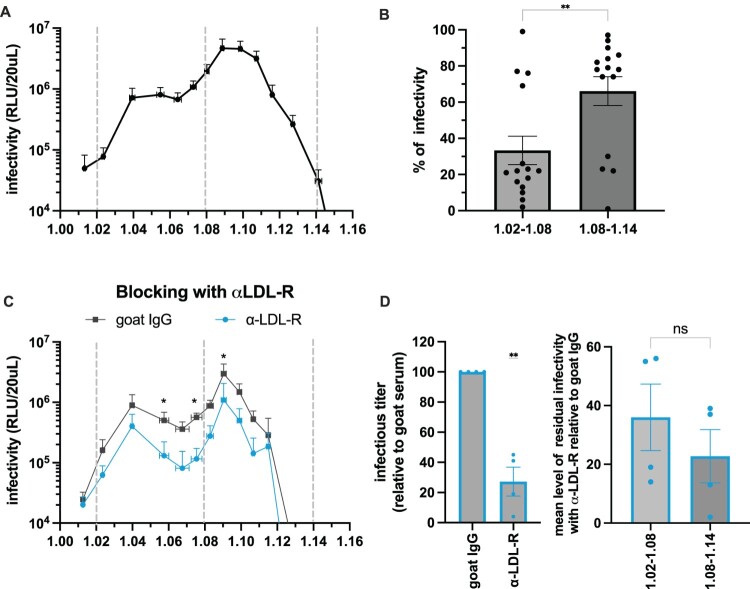
CCHFV particles have a heterogeneous profile of density. (A) Density-gradient analysis of CCHFVmg-nLuc particles harvested in serum-free medium at 72 h post-transfection of Huh-7.5 cells. Particles were layered on top of a 3–40% continuous iodixanol gradient and were centrifuged for 16 h at 32,000 rpm in a SW41 swinging rotor at 4°C. 15 fractions were collected and used for infection assays of Huh-7.5 cells pre-transfected with NP + L expressing plasmid. Infected cells were harvested at 24 h post-infection, and infectivity was determined by measurement of nanoluc signals. The graph displays the mean infectivity in each fraction from different gradients. Results from 15 independent experiments. (B) The value of infectivity from different gradients was regrouped into two density categories, low-density fractions (1.02–1.08) and high-density fractions (1.08–1.14), and were normalized to the total infectivity in order to assess the percentage of infectivity in each category. Results display the mean of percentages of infectivity (n = 15). Unpaired t-test. (C) Gradients from (A) were used for infection of Huh-7.5 cells pre-incubated for 1 h at 37°C with 4 μg/mL of LDL-R antibody (blue) or control goat IgG (grey) before infection. Infection was performed in the presence of fresh antibody. Cells were harvested 24 h post-infection, and infectivity was determined by measurement of nanoluc signal. The results display the mean infectivity in each fraction from 4 different gradients. Paired ratio t-test. (D) (Left) level of total residual infectivity with anti-LDL-R relative to goat IgG from (C) was evaluated by summing the infectivity of each fraction; (right) level of residual infectivity with anti-LDLR relative to goat IgG from (C) was evaluated for each fraction and the mean of this level was determined for the two categories: low-density fractions (1.02–1.08) and high-density fractions (1.08–1.14). n = 4. Paired ratio t-test. Data are represented as means ± SEM. For (B) and (D), each dot represents data from individual gradients.

### Production and infection assays with HAZV particles

The procedure was performed as described in Ritter et al. [7].

### Production and titration of full-length CCHFV particles

The procedure was performed as described in Ritter et al. [7].

### Iodixanol density gradient of CCHFVmg particles

One millilitre of supernatant of CCHFVmg-nLuc particles was layered on top of a 3–40% continuous iodixanol gradient (Optiprep; Axis-Shield). Gradients were centrifuged for 16 h at 126,444 xg in a SW41 swinging rotor at 4°C using an Optima L-90 K Beckmann centrifuge. Fifteen fractions of 750 µL were collected from the top and analysed for virus infectivity and viral RNA copies. The density of the fractions was determined by refractometry analysis. For infectivity, Huh-7.5 stably expressing firefly luciferase (Fluc) and pre-transfected with pCAGGS-V5-L and pCAGGS-NP were transduced with two dilutions of fractions. At 24 h post-transduction, cells were lysed with passive lysis buffer (Promega) for 10 min at room temperature, and the luciferase signal was measured using the Nano-Glo® Dual-Luciferase® Reporter Assay System (Promega). For each dilution, technical replicates were performed. The viral titre was determined with dilutions within a linear range of NanoLuc signals.

For neutralization assays with anti-apoE or anti-apoB sera or soluble LDL-R/LRP8, fractions were incubated for 1 h at room temperature with anti-apoB serum, anti-apoE serum, or control goat serum diluted 1/100 or with soluble LDL-R, soluble LRP8, or soluble CD81-LEL at 5 ug/mL and then added to Huh-7.5 cells pre-transfected with pCAGGS-V5-L and pCAGGS-NP. At 3 h post-infection or post-transduction, the media were replaced with DMEM, 10% FBS. Twenty-four hour post-transduction, cells were harvested and treated as described above for evaluation of transduction titre.

For treatment with lipoprotein lipase (LPL), CCHFVmg particles were incubated with 25 μg/mL of LPL (Sigma-Aldrich, L2254) or PBS for 1 h at 37°C.

The levels of apoE or apoB in fractions from density gradients were assessed by ELISA (Mabtech 3712-1HP and 3715-1HP) according to the manufacturer’s protocol.

### Knockdown of apoB or DGAT-1

apoB shRNAs (TRCN0000003739) or DGAT1 shRNAs (TRCN0000036149 and TRCN0000036151) were ordered from Sigma-Aldrich. To package shRNA-expressing lentiviral vectors, HEK293T cells were seeded in 10-cm plates and were transfected with 8 µg of the pLKO construct or pLKO apoB construct, 8 µg of the HIV packaging construct psPAX2, and 2.7 µg of the VSV-G glycoprotein construct phCMV-G. The vector particles were collected 48 h post-transfection with 0.45 µm filters, and aliquots were kept at –80°C for further use. The infectious titres were determined on 293T cells by VCN analysis. To downregulate apoB or DGAT-1 in Huh-7.5 cells, the shRNA lentiviral vectors were used at an MOI of 5. At 16 h post- transduction, cells were used for further transfections to produce CCHFVmg particles as described above or were infected with HAZV to produce HAZV particles for 3 days.

### Blocking with anti-LDL-R antibody

The procedure of blocking was done as described previously [7] with two dilutions of viral fractions from density gradients.

### Neutralization assays with anti-apoB or anti-apoE sera

Serial dilutions of inoculate were incubated for 1 h at room temperature with anti-apoB serum, anti-apoE serum, or control goat serum diluted 1/100 and then added to Huh-7.5 cells grown in OptiMEM as described previously [7]. In the case of CCHFVmg particles, Huh-7.5 cells were pre-transfected with pCAGGS-V5-L and pCAGGS-NP as described above. All the infection/transduction assays were performed with serial dilutions. At 3 h post-infection or post-transduction, media were replaced with DMEM, 10% FBS. Twenty-four hour post-infection or post-transduction, cells were harvested and treated as described above for evaluation of infectious/ transduction titre.

### Co-immunoprecipitation assay

The procedure used was performed as described [7] using anti-apoB serum.

### Drug treatment

For CCHFVmg particles, Huh-7.5 cells were pretransfected with pCAGGS-NP, pCAGGS-V5-L, and pCAGGS-GPC. At 24 h post-transfection, cells were infected with CCHFVmg-GFP particles at MOI 1. At 3 h post-infection, medium was changed for OptiMEM with drug/vehicle at the concentrations indicated in the Supplementary Table 1, unless otherwise specified. Supernatants were harvested at 24 or 48 h post-treatment for the determination of the levels of infection by flow cytometry. Cell viability was assessed in parallel using Cytotox-Glo Cytotoxicity Assay (Promega) according to the manufacturer’s protocol. Conditions reducing cell viability to 80% were considered cytotoxic.

For wild-type (WT) CCHFV, infections of Huh-7.5 cells were performed with a MOI of 0.01. At 1 h post-infection, the medium was changed for OptiMEM with the indicated molecules. At 24 h post-infection, supernatants were collected and lysed with Trizol. Infected cells were also lysed with Trizol. RNAs were extracted according to the manufacturer’s protocol, and the level of viral RNA, reflecting the level of infection, was determined by RT-qPCR (see below). For evaluation of the infectious titre, several dilutions of supernatants were used to infect naïve Huh-7.5 cells and proceed as described for neutralization assays. Infection of PHH was performed with a MOI of 0.1-0.2, and all the experiments were performed as for Huh-7.5 cells except that the culture medium was changed to PHH medium.

### RNA extraction and RT-qPCR analysis

The procedure was adapted from [7,17]. All sequences of primers are provided in Supplementary Table 1. For CCHFV, the RNA quantification was performed with primers targeting the L untranslated regions (UTR). For HAZV, the RNA quantification was done with the primers targeting the S segment. For titration of WT CCHFV, viral RNA levels were normalized with respect to glyceraldehyde-3-phosphate dehydrogenase (GAPDH) RNA levels. For signal of crude supernatants as well as immunoprecipitation (IP) samples, an exogenous RNA from the linearized Triplescript plasmid pTRI-Xef (Invitrogen) was added into the supernatant prior to extraction and quantified with specific primers.

### Western blot analysis

The procedure was adapted from [7,18]. To detect the expression of apoB and Actin, samples were denatured at 95°C for 5 min in a reducing loading buffer and electrophoresed on 6% polyacrylamide gels. For the detection of Gc and apoB in IP samples, extracted proteins were processed in a non-reducing loading buffer and electrophoresed on 10% polyacrylamide gels.

### Immunofluorescence analysis

Huh-7.5 cells were grown in six-well plates containing uncoated glass coverslips and were transfected with pCAGGS-V5-L, pCAGGS-NP, and pCAGGS-GPC expression plasmids. The transfection media was replaced after 6 h post-transfection, and infected with CCHFVmg particles at 24 h post-transfection. Forty-eight hours post-transfection, cells were fixed with 4% paraformaldehyde (PFA) for 15 min at room temperature. Following three washes with PBS, cells were permeabilized with 0.1% Triton X-100 for 7 min and then were saturated with 3% bovine serum albumin (BSA)/PBS for 20 min. Cells were incubated for 1 h at room temperature with anti-ApoB serum (AB742, 1/100) and anti-Gc (11E7, 1/200) primary antibody diluted in PBS/1% BSA. After three washes with PBS/1% BSA, cells were further incubated with Alexa Fluor-conjugated secondary antibodies (anti-Goat-AF555 and anti-Mouse-AF647 1/2000, respectively; Thermo Fisher) in PBS/1% BSA. After three washes with PBS, nuclei were stained with Hoechst 33342 (Molecular Probes), and the coverslips were mounted with Mowiol 40-88 (Sigma-Aldrich). The slides were examined using a confocal microscope (LSM-800, Zeiss) equipped with a 63X objective. Pearson’s correlation coefficients were calculated using FIJI (JACoP) and were calculated on 10 cells from two separate experiments and expressed as mean ± SEM.

### Electron microscopy

CCHFVmg particles produced without serum or in the presence of 10% HS were concentrated by Vivaspin 100 kDa before purification by ultracentrifugation (2 h, 96,589 xg, 4°C) without a sucrose cushion. The procedure for negative staining of particles and the analysis by transmission electron microscopy (JEM-1400Plus) was performed as described in [7].

### Statistical analysis

Significance values were calculated by applying tests indicated in the figure legends using the GraphPad Prism 10 software (GraphPad Software, USA). For statistical analysis, a *P*-value of 0.05 or less was considered significant. Data are presented as mean ± standard error of the mean (SEM), and results of the statistical analysis are shown as follows: ns, not significant (*P* > 0.05); *, *P* < 0.05; **, *P* < 0.01; and ***, *P* < 0.001.

## Results

### CCHFV particles are heterogeneous and detected within a large range of densities

The recruitment of lipoprotein components onto CCHFV particles may stochastically generate different virion subpopulations during production. To address this question, we examined the density of CCHFVmg-nLuc particles produced in Huh-7.5 hepatocyte-derived cells using buoyant density gradient analyses. We found a strong heterogeneity of infectious particles, ranging from densities <1.02 to 1.14 (Figure 1A). The profile of viral particles in the gradient exhibited a major peak at ca. 1.09 and a marked shoulder at 1.04–1.07, suggesting that there could be at least two major populations of infectious CCHFV particles that overlap. About 33% of infectious particles were comprised between densities of 1.02 and 1.08 (Figure 1B) and were therefore designated low-density CCHFVmg particles, whereas ca. 67% were found in higher densities (1.08–1.14).

To address whether either low- or high-density CCHFVmg particles use the same receptor for cell entry, we examined the dependency of viral particles of each density on LDL-R using a blocking antibody or, alternatively, soluble LDL-R [7]. We found that such treatment significantly reduced the infectivity of particles from all fractions of the gradient, indicating that both low- and high-density CCHFVmg particles share the same dependency on LDL-R (Figure 1C–D and Supplementary Figure 2A–B).

Altogether, these results indicated that CCHFVmg particles display a heterogeneous composition, leading to different infectious subpopulations that use LDL-R for entry into cells.

### ApoB promotes CCHFV infectivity through specific association with viral particles

**Figure 2.**
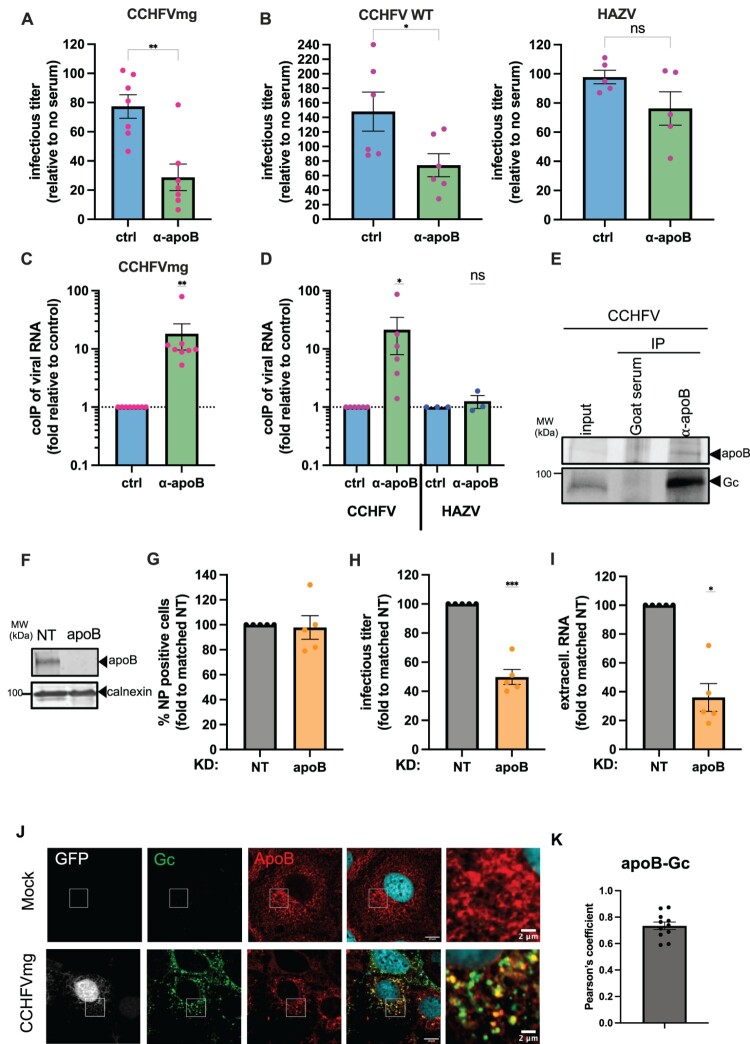
apoB is associated with CCHFV particles and promotes infectivity. (A) CCHFVmg-GFP particles were incubated for 1 h at room temperature with anti-apoB serum or with a control goat serum (ctrl) before infection of Huh-7.5 cells pre-transfected with NP + L expression plasmids. Cells were harvested at 24 h post-infection, and infectivity was determined by flow cytometry. Results from 7 independent experiments. Unpaired t-test. (B) Same experiment with wild-type (WT) CCHFV (left) or HAZV (right). Media was removed at 1 h post-infection, and cells were lysed at 24 h post-infection for CCHFV or fixed for HAZV. The infectivity was quantified by RT-qPCR for CCHFV or by flow cytometry for HAZV. Results from 6 and 5 independent experiments, respectively. Unpaired t-test. (C) CCHFVmg-GFP particles were immunoprecipitated with an anti-apoB serum or with a control goat serum (ctrl). The levels of CCHFV minigenome co-immunoprecipitated with anti-apoB serum vs. control serum were quantified by RT-qPCR. Results from 8 independent experiments are presented as fold enrichment with anti-apoB serum relative to control serum. The Wilcoxon signed-rank test was used to compare the value of anti-apoB serum with a theoretical value of 1. (D) Same experiment using full-length CCHFV particles or HAZV particles. Results from 6 (CCHFV) or 3 (HAZV) independent experiments are presented as fold enrichment with anti-apoB serum relative to control serum. The Wilcoxon signed-rank test was used to compare the value with anti-apoB serum to a theoretical value of 1. (E) Immunoprecipitation of CCHFV particles was analyzed by Western blot using anti-apoB serum and Gc antibodies. (F) Intracellular levels of apoB as assessed by Western blots of control Huh-7.5 cells (NT) or of Huh-7.5 cells transduced with an apoB-targeting shRNA-expressing lentiviral vector. Representative image of 3 independent experiments. (G) Huh-7.5 cells described in (F) were used for the production of CCHFVmg particles. Percentage of CCHFV NP in transfected cells assessed by flow cytometry. (H) Infectious titres of CCHFVmg particles produced in Huh-7.5 cells, as described in (F), were assessed by flow cytometry. (I) Levels of secreted CCHFV RNA of CCHFVmg particles assessed by RT-qPCR. For (G – I), results from 5 independent experiments are expressed as fold relative to NT. One-sample t-test. (J) Intracellular localization of apoB (red) or Gc (green) in Huh-7.5 cells expressing NP + L + GPC and infected with CCHFVmg-GFP particles or in Mock cells as assessed by confocal microscopy. Scale bars represent 2 μm. (K) Pearson’s correlation coefficient of colocalization between Gc and apoB. Data are represented as means ± SEM. For (A–I), each dot in the graphs corresponds to the value of an individual experiment.

The acquisition of a low density for CCHFV particles (Figure 1) could reflect their association with lipoproteins, such as very low-density lipoproteins (VLDL) and low-density lipoproteins (LDL), or at least lipoprotein components, such as apolipoprotein E (apoE) [7]. As apolipoprotein B (apoB), a non-exchangeable apolipoprotein, is the major structural protein component of VLDLs and LDLs, which are very abundant in human serum (HS), we wondered if CCHFV particles could be associated with apoB. First, to address the possibility that apoB may contribute to CCHFV infectivity; we incubated CCHFV particles with anti-apoB serum for 1 h before infection. We found that, compared to the control condition, CCHFVmg-GFP infectivity was effectively inhibited by anti-apoB serum by over 70% (Figure 2A). We then sought to extend this result to WT CCHFV, which was handled in the Jean Mérieux BSL4 (Lyon, France), and to Hazara virus (HAZV), which also belongs to the *Orthonairovirus* genus. We found that anti-apoB serum inhibited WT CCHFV particles (Figure 2B), validating the above findings using CCHFVmg particles, but did not significantly inhibit infection with HAZV particles (Figure 2B).

These results might be explained by the efficient recruitment of apoB to the surface of CCHFV particles. Thus, to address this question, we used anti-apoB serum to capture viral particles, and we used RT-qPCR assays on viral RNA to quantify the particles that were collected following immunoprecipitation. Remarkably, compared to control serum, we detected a ca. 20-fold enrichment of CCHFV RNAs with anti-apoB serum for both CCHFVmg-GFP and WT CCHFV particles (Figure 2C–D). Yet, in line with the results of apoB neutralization, we failed to capture HAZV particles (Figure 2D), indicating that apoB, like apoE [7], is selectively associated with CCHFV particles. Furthermore, we confirmed the association of WT CCHFV particles with apoB, as we could also co-capture CCHFV Gc proteins with anti-apoB serum (Figure 2E).

Next, we sought to address the possibility that apoB could play a role in CCHFV assembly. We therefore down-regulated apoB in CCHFVmg-GFP producer cells upon transduction with an apoB shRNA-expressing lentiviral vector, which induced a robust loss of apoB expression (Figure 2F) before allowing CCHFVmg production. While apoB knock down (KD) did not impair the level of expression of CCHFV NP in these producer cells (Figure 2G), it resulted in a loss of production of CCHFVmg infectious particles by up to 55% compared to non-transduced cells (Figure 2H), in agreement with the results of apoB neutralization (Figure 2A–B). Furthermore, we found that the amounts of CCHFV RNA in the supernatants of apoB KD producer cells were reduced in a similar proportion of up to 65% (Figure 2I), suggesting that apoB facilitates the assembly/secretion of CCHFV particles, which results in increased infectious titres. In concurrence with this possibility, we observed a strong colocalization between apoB and CCHFV Gc glycoprotein in CCHFVmg producer cells (Figure 2J–K), suggesting that the assembly of CCHFV particles and the synthesis of apoB-containing lipoproteins could be related events. Finally, in line with the results of apoB neutralization (Figure 2B) and immunocapture (Figure 2D), we found that HAZV particle production was not prevented by apoB KD in virus producer cells (Supplemental Figure 3).

These results indicated that CCHFV usurps apoB for the production of infectious particles, which leads to the incorporation of apoB on their surface.

### ApoB and apoE promote infectivity of low-density CCHFV particles

**Figure 3.**
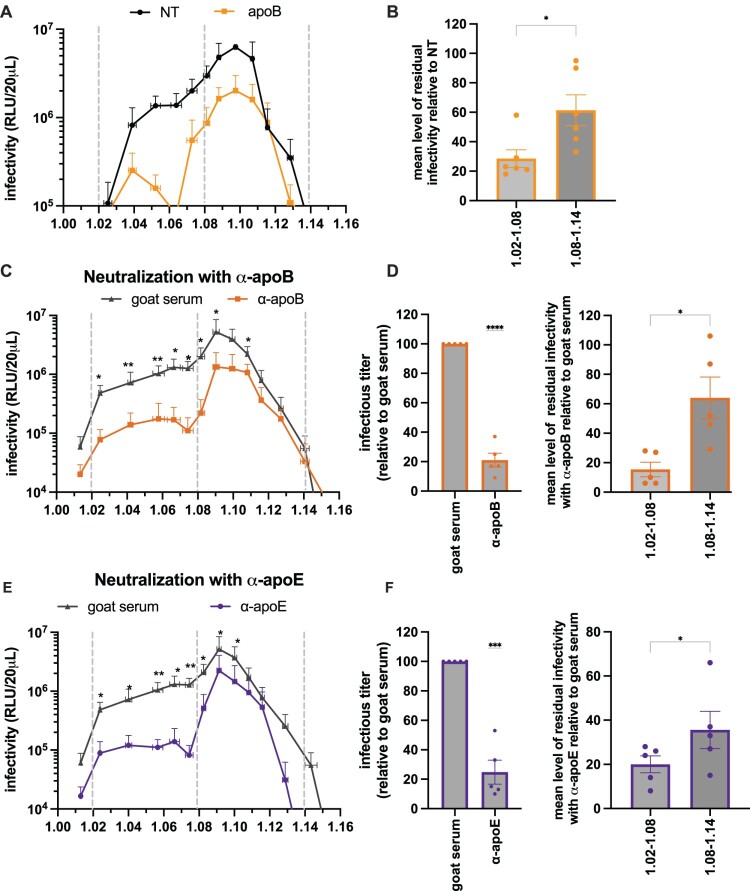
apoB is more associated with low-density CCHFV particles. (A) CCHFVmg-nLuc particles produced in control Huh-7.5 cells (NT, black) or in cells knock-down for apoB expression (apoB, orange) were used for density gradient analysis. The results display the means of infectivity in each fraction from the different gradients. Results are from 6 independent experiments. Paired ratio t-test. (B) Level of residual infectivity upon apoB KD was evaluated for each fraction, and the mean level was determined for two categories: low-density fractions (1.02–1.08) and high-density fractions (1.08–1.14). Results from 6 independent experiments. Paired ratio t-test. (C) Fractions from gradients as described in Figure 1A were incubated for 1 h at room temperature with anti-apoB serum (orange) or with control goat serum (grey) before infection. Cells were harvested at 24 h post-infection, and infectivity was determined by measurement of nanoluc signals. The results display the means of infectivity in each fraction from 5 different gradients. Paired ratio t-test. (D) (Left) level of total residual infectivity with anti-apoB serum relative to goat serum from (C) was evaluated by summing the infectivity of each fraction; (right) level of residual infectivity with anti-apoB serum relative to control goat serum from (C) was evaluated for each fraction and the mean level was determined for two categories: low-density fractions (1.02–1.08) and high-density fractions (1.08–1.14). Results from 5 different gradients. Paired ratio t-test. (E) Same as (C) with anti-apoE serum (violet). The results display the mean of infectivity from different 5 gradients. Paired ratio t-test. (F) (Left) level of total residual infectivity with anti-apoE serum relative to goat serum from (C) was evaluated by summing the infectivity of each fraction; (right) level of residual infectivity with anti-apoE serum relative to control goat serum from (E) was evaluated for each fraction and the mean level of residual infectivity was determined for two categories: low-density fractions (1.02–1.08) and high-density fractions (1.08–1.14). Results from 5 different gradients. Paired ratio t-test. Data are represented as means ± SEM. For (B), (D), and (F), each dot represents data from individual gradients.

We sought to investigate whether apoB expression in CCHFV producer cells could influence the density of released viral particles. Notably, we found that compared to control cells, apoB KD cells produced significantly less CCHFVmg-nLuc infectious particles of low density (1.02–1.08), which decreased the proportion of these low-density infectious particles by 3.5-fold on average, while the production of high-density CCHFV particles was decreased by only 1.6-fold (Figure 3A–B). This resulted in a clear distinction between two subpopulations of CCHFVmg particles of low- vs. high-density particles, suggesting that CCHFV exploits apoB expression to induce the production of infectious particles of low density.

Next, to address whether apolipoproteins play a role subsequent to their incorporation into CCHFV particles, we used anti-apoB or anti-apoE sera to treat CCHFV particles recovered from density gradients and assessed their resulting infectivity. We found that the low-density viral particles were neutralized by ca. 6.7-fold (range: 5–11-fold, depending on the fractions) by the anti-apoB serum, while the infectivity of the population of high density was reduced by 1.6-fold (Figure 3C–D). Likewise, we found that the low-density viral particles were neutralized by ca. 5-fold (range: 5–15-fold, depending on the fractions) by the anti-apoE serum, while the infectivity of the population of high density was reduced by 2.8-fold (Figure 3E–F). To confirm this, we tested the neutralization of CCHFVmg with soluble LRP8, which can inhibit viral infection via binding to apoE [8]. We found that only low-density fractions were sensitive, though moderately, to soluble LRP8 (Supplemental Figure 4A–B).

Together, our findings indicated that apoB and apoE recruited on CCHFV particles promote the infectivity of viral particles with a low density.

### Composition of the extracellular medium influences the density of CCHFV particles

Having noticed that the low-density fractions correspond to fractions where apoE and apoB can be readily detected (Supplemental Figure 5), we sought to investigate the nature of the low-density CCHFV particles.

**Figure 4.**
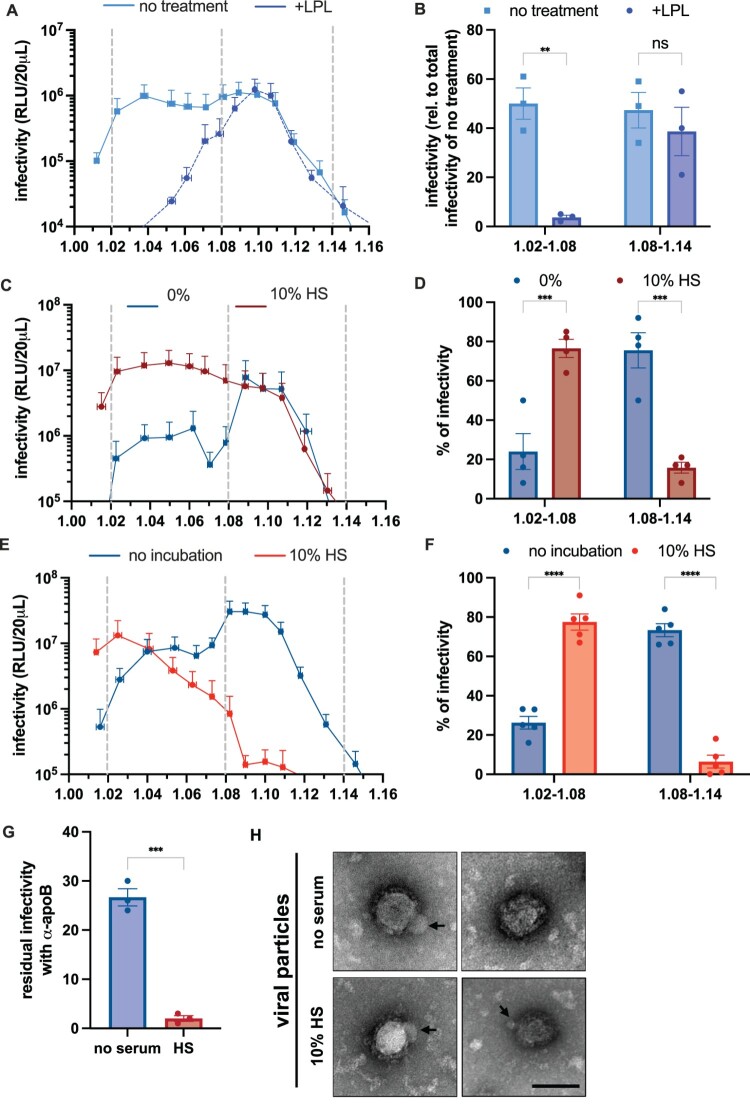
The density of CCHFV particles is influenced by the composition of extracellular medium. (A) CCHFVmg-nLuc particles produced in serum-free medium were incubated with lipoprotein lipase (LPL, dark blue) or without lipase (light blue) for 1 h at 37°C before analysis in density gradients. The results display the means of infectivity in each fraction from 3 different gradients. (B) Infectivity values from gradients of (A) were regrouped into two categories, low-density fractions (1.02–1.08) and high-density fractions (1.08–1.14), and were normalized to the total of infectivity of the condition without LPL. Results from 3 independent experiments. Two-way ANOVA test with Šídák’s multiple comparisons test. (C) Same experiment as described in Figure 1A of density gradient analysis of CCHFVmg-nLuc particles produced either in serum-free medium (blue) or with 10% human serum (red). The results display the means of infectivity in each fraction from 4 different gradients. (D) Infectivity values from gradients of (B) were regrouped into two categories (low-density from 1.02 to 1.08, and high-density from 1.08 to 1.14) and normalized to the total of infectivity. Results display as the mean percentages of infectivity in either category of 4 independent experiments. Two-way ANOVA test with Šídák’s multiple comparisons test. (E) CCHFVmg-nLuc particles produced in serum-free medium were left untreated (no incubation, blue) or incubated for 6 h at 37°C with 10% human serum (red) before density gradient analysis. The results display the means of infectivity in each fraction from 5 different gradients. (F) Infectivity values from (E) were respectively regrouped into two categories: low-density fractions (1.02–1.08) and high-density fractions (1.08–1.14), and were normalized to the total of infectivity. Results display the mean percentages of infectivity in either category of 5 independent experiments. Two-way ANOVA test with Šídák’s multiple comparisons test. (G) CCHFVmg-nLuc produced without serum (blue) or in the presence of 10% HS was used for anti-apoB serum neutralization assays as described in Figure 2A. Results from 3 independent experiments. Unpaired t-test. (H) Representative electron microscopy images of CCHFVmg particles produced without or with 10% HS. Scale bars represent 100 nm. Data are represented as means ± SEM. For (B), (D), and (F), each dot represents an individual gradient.

First, we thought that since apoB is a non-exchangeable apolipoprotein detected at the surface of LDLs and VLDLs, its binding to CCHFV particles should be accompanied by apoB-associated neutral lipids. Therefore, to address this hypothesis, we treated CCHFV particles recovered from density gradients with lipoprotein lipase (LPL), an enzyme responsible for the hydrolysis of triglycerides contained in VLDLs and LDLs, and we assessed their resulting infectivity. We found that LPL-treatment inhibited the infectivity of the low-density, though not the high-density, CCHFVmg-nLuc particles, suggesting that the infectivity of the former depends on neutral lipid-associated apoB (Figure 4A–B).

Second, since CCHFVmg particles were produced from Huh-7.5 cells grown in serum-free conditions to assess better how producer cells influence their properties, we wondered whether the composition of the extracellular environment could also influence virion density. Surprisingly, when we produced CCHFVmg-nLuc particles in the presence of 10% human serum (HS), we observed an inversion of the profile of repartition of viral particles along the gradient (Figure 4C), with ca. 3.2-fold more infectious particles in the low-density fractions and ca. 4.8-fold less infectious particles in the high-density fractions, as compared to CCHFVmg particles produced in serum-free medium (76.5% vs. 24% and 15.6% vs. 75.5%, respectively) (Figure 4D). This suggested that HS could influence the density and, hence, the composition of CCHFV particles.

Third, we reasoned that enveloped viral particles can be considered lipid carriers and, thus, may be reshaped through interactions with factors regulating lipoprotein metabolism. In this respect, their association with lipoprotein components, such as apolipoproteins, could occur either during viral production, e.g. through co-optation of the lipoprotein biosynthesis pathway, as shown before (Figure 2, Figure 3), or, alternatively, after egress in the extracellular medium. To address whether the latter hypothesis could explain the above results (Figure 4C–D), we mixed CCHFVmg-nLuc particles produced in serum-free medium with HS, which provides a full lipoprotein environment. Strikingly, when we incubated CCHFV particles for 1 h at 37°C with 10% HS, we observed a peak of infectious particles at a density of around 1.025 (Figure 4E), with ca. 95% of the viral particles shifted at low densities below 1.08 (Figure 4F).

Finally, as suggested by the results in Figure 3C–D and Figure 2C–E, we surmised that CCHFVmg-nLuc particles produced in HS could be particularly sensitive to anti-apoB serum. Using the same procedure as in Figure 3A, we found that while untreated CCHFVmg particles were neutralized by ca. 70% by anti-apoB serum, neutralization levels reached up to 98% when they were produced in 10% HS (Figure 4G), corroborating the idea that the acquisition of low-density was correlated with the association of apoB with the viral particles.

Altogether, these results indicated that secreted CCHFV particles could evolve in the extracellular medium by associating with human serum components, such as apoB, which shifts infectious particles toward lower densities. It should be noted that the electron microscopy (EM) analysis of CCHFVmg particles produced in serum-free medium revealed some viral particles having a putative association with small components resembling lipoproteins (Figure 4H, arrows). Production in medium containing HS did not reveal any differences in the shape of the viral particles or any aggregation with serum components (Figure 4H); however, this does not rule out the existence of potentially weak associations, which could be lost during purification.

### Host factors of the lipoprotein synthesis pathway and lipid metabolism regulate production of CCHFV infectious particles

**Figure 5.**
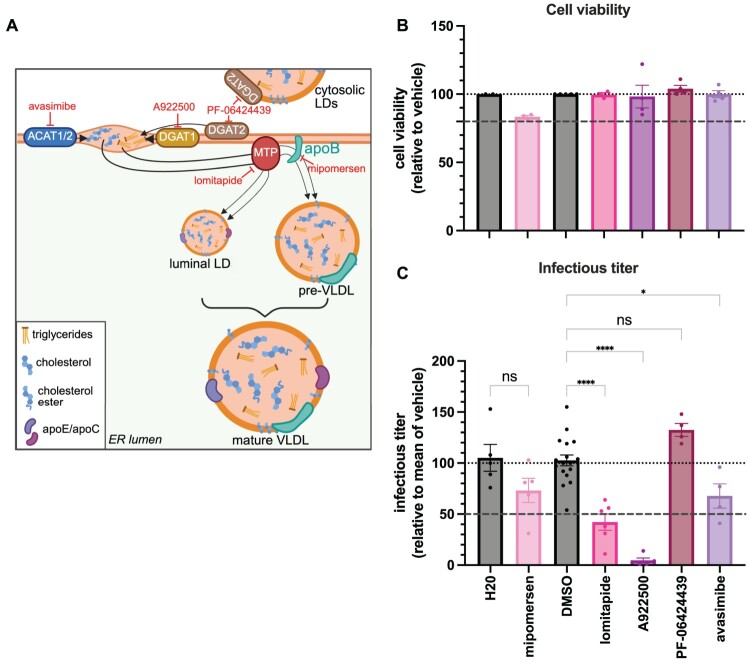
Molecules targeting lipoprotein metabolism are antiviral. (A) Schematic illustration of very-low-density lipoprotein (VLDL) biogenesis in the endoplasmic reticulum (ER) lumen. Triglycerides (orange), cholesterol (blue), and cholesterol esters (blue clusters) are synthesized and packaged into VLDLs. Key steps include lipid droplet (LD) formation, apolipoprotein B (apoB) recruitment, and microsomal triglyceride transfer protein (MTP)-mediated lipidation, resulting in the formation of pre-VLDL and mature VLDL particles. Apolipoproteins E and C (apoE/apoC, purple) are incorporated during maturation. Inhibitors targeting specific steps of this pathway are indicated in red: avasimibe (ACAT1/2 inhibitor), A922500 and PF-06424439 (DGAT1 and DGAT2 inhibitors, respectively), lomitapide (MTP inhibitor), and mipomersen (apoB antisense oligonucleotide). (B) Huh-7.5 cells were treated with mipomersen, lomitapide, A922500, PF-06424439, and avasimibe, using the concentrations specified in Supplementary Table 1, or with H20 and DMSO (as control vehicles). Cell viability was assessed at 24 h after treatment. Dotted lines indicate reference thresholds (80%, 100%) and are included to facilitate interpretation of the data. (C) Same cells from (B) seeded in a 12-well plate and infected with CCHFVmg particles with MOI = 1 at 24 h post-transfection. 3 h after infection, cells were treated with inhibitors as in (B). 24 h after infection, supernatants were collected and used for titration in Huh-7.5 cells pre-transfected with pCAGGS-NP and pCAGGS-V5-L plasmids. Infectious viral titres from cells treated with the indicated inhibitors, normalized to the mean values obtained with the control vehicle. One-way ANOVA with Šídák’s multiple comparisons test. Dotted lines indicate reference thresholds (50%, 100%) and are included to facilitate interpretation of the data. Data are presented as means ± SEM. Each dot in the graphs corresponds to the value of an individual experiment. Results from 4–6 independent experiments.

The above results, combined with the fact that apoB is required for the production of lipoproteins, suggested that the lipoprotein synthesis pathway could be hijacked by CCHFV. To address this question and to identify the involved host factors, we targeted key enzymes of this pathway using specific inhibitors (Figure 5A). We first selected mipomersen, an antisense oligonucleotide inhibitor of apoB; lomitapide, an inhibitor of the microsomal triglyceride transfer protein (MTP), which mediates the transfer of neutral lipids to nascent apoB to form pre-VLDLs and luminal lipid droplets. We also extended these assays with inhibitors targeting the formation of triglycerides and cholesterol esters: A922500 and PF-06424439, inhibitors of the diacylglycerol O-acyltransferases DGAT-1 and DGAT-2, respectively, two enzymes responsible of the last step in the synthesis of triglycerides; and avasimibe, an inhibitor of the acyl-coenzyme A:cholesterol acyltransferases ACAT-1 and ACAT-2, two enzymes responsible of the last step in the synthesis of cholesterol esters. Note that these inhibitors not only impair lipoprotein metabolism but also more generally modulate lipid metabolism. We used the doses of either inhibitor that were shown not to be toxic in our experimental conditions (Figure 5B).

We found that mipomersen had a mild though significant effect on the production of infectious CCHFVmg-GFP particles (Figure 5C), consistent with the results obtained in apoB KD cells. In contrast, lomitapide treatment led to a ca. 3-fold reduction, confirming the involvement of the lipoprotein biogenesis pathway in CCHFV infection. DGAT-1 inhibition caused a pronounced impairment of CCHFV infectivity, reducing particle production by more than 21-fold, whereas DGAT-2 inhibition slightly increased particle production, highlighting the importance of triglyceride synthesis in viral replication. Finally, inhibition of cholesterol ester synthesis with an ACAT-1/2 inhibitor had only a mild effect, indicating that cholesterol ester production plays a limited role in CCHFV production.

Overall, our results suggested that CCHFV hijacks the metabolism of host lipoproteins as well as the metabolism of triglycerides, more generally, to enhance its infectivity and led us to identify key enzymes that regulate the production of CCHFV infectious particles.

### Targeting liver metabolism can be used as an antiviral strategy

**Figure 6.**
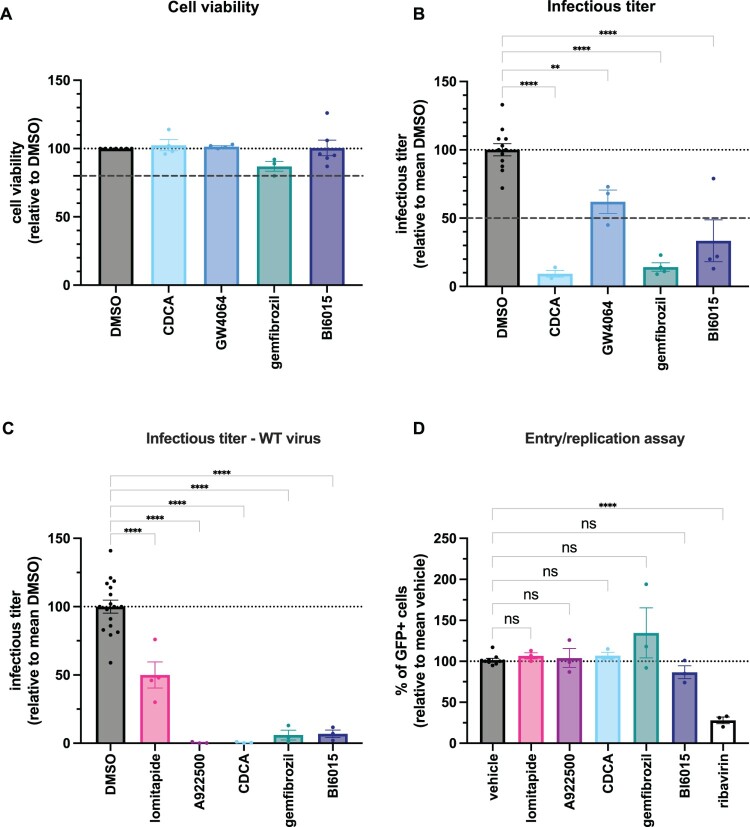
Molecules targeting liver metabolism are antiviral. (A) Huh-7.5 cells were treated with bile acid or lipid metabolism modulators, including CDCA (chenodeoxycholic acid), GW4064 (FXR agonist), gemfibrozil (PPARα agonist), and BI6015 (HNF4α inhibitor), using the concentrations specified in Supplementary Table 1. Cell viability was measured relative to the DMSO vehicle control. Dotted lines indicate reference thresholds (80%, 100%) and are included to facilitate interpretation of the data. (B) Same cells as in (A) were seeded in a 12-well plate and infected with CCHFVmg-GFP particles. 3 h after infection, cells were treated with inhibitors as in (A). 24 h after infection, supernatants were collected and used for titration in Huh-7.5 cells, pre-transfected with pCAGGS-NP and pCAGGS-V5-L plasmids. Infectious viral titre of virus produced in cells treated with the inhibitors, normalized to the mean of values of DMSO. One-way ANOVA with Dunnett’s multiple comparison. Dotted lines indicate reference thresholds (50%, 100%) and are included to facilitate interpretation of the data. For (A) and (B), results from 3–6 independent experiments. (C) Huh-7.5 cells were seeded in a 12-well plate and infected with full-length CCHFV particles at MOI = 0.01. 1 h after infection, cells were treated with inhibitors in (A). 24 h after infection, supernatants were collected and used for titration in Huh-7.5 cells. Infectious titre of virus produced in cells treated with the inhibitors normalized to the mean of values obtained with DMSO. One-way ANOVA with Dunnett’s multiple comparison. The dotted line indicates reference thresholds (100%) and is included to facilitate interpretation of the data. Results from 3–4 independent experiments. (D) Huh-7.5 cells were pre-transfected with pCAGGS-NP and pCAGGS-V5-L plasmids before infection with CCHFVmg-GFP particles (MOI = 1) at 24 h post-transfection. 3 h after infection, cells were treated with inhibitors as in (C) or with ribavirin. 24 h after infection, cells were harvested and the level of GFP was assessed by flow cytometry. Results from 3 independent experiments. One-way ANOVA with Dunnett’s multiple comparison. Data are shown as means ± SEM. Each dot in the graphs corresponds to the value of an individual experiment.

The above findings suggested that lipoprotein metabolism and triglyceride metabolism could be suitable targets for a host-targeting antiviral strategy. Lipoprotein synthesis, and more generally lipid metabolism, is regulated in hepatocytes at different levels via the activation/blocking of multiple factors, such as farnesoid X receptor alpha (FXRα), hepatocyte nuclear factor 4 alpha (HNF4α), and peroxisome proliferator-activated receptor alpha (PPARα), all of which can be modulated by small molecules. We therefore tested: an inhibitor of HNF4α, BI6015; the natural ligand of FXRα, chenodeoxycholic acid (CDCA), as well as a synthetic ligand of FXRα, GW4064; and an agonist of PPARα, gemfibrozil. Interestingly, using non-toxic doses of either inhibitor (Figure 6A), we observed an over 11-fold impairment of production of CCHFVmg-GFP upon CDCA treatment, while GW4064 treatment showed only a moderate effect. We also observed a robust inhibition of CCHFVmg-GFP production with BI6015 and Gemfibrozil with a 3- and 7-fold decrease of infectivity, respectively (Figure 6B).

Based on these results, we next tested the most effective molecules for their antiviral activity on WT CCHFV. We selected the molecules that had an inhibitory effect higher than 50% on CCHFVmg infectivity (Figure 5C and Figure 6B), i.e. lomitapide, A922500, CDCA, BI6015, and gemfibrozil. Importantly, we could confirm the strong antiviral effects of A922500 and CDCA with a 250-fold and 189-fold decrease in the production of infectious particles, respectively, as well as the effects of lomitapide, BI6015, and gemfibrozil with a 2-fold, 14-fold, and 17-fold decrease, respectively (Figure 6C).

The above results indicated that small-molecule inhibitors targeting lipid metabolism can prevent CCHFV production. To determine whether either molecule can inhibit viral entry/replication or assembly/secretion, we infected cells that only allow entry and replication, then treated them with the drugs. We found that the inhibitors did not change the levels of CCHFVmg-infected cells relative to non-treated cells, in contrast to ribavirin, a known inhibitor of CCHFV replication (Figure 6D). These results ruled out the possibility that the above-mentioned molecules prevent entry and/or replication/translation and suggested that they act on the assembly and/or secretion of viral particles.

### DGAT-1 inhibitors effectively impede the infectivity of the CCHFV virus at various levels

Next, we sought to characterize better the effects associated with DGAT-1 inhibition on virus production.

**Figure 7.**
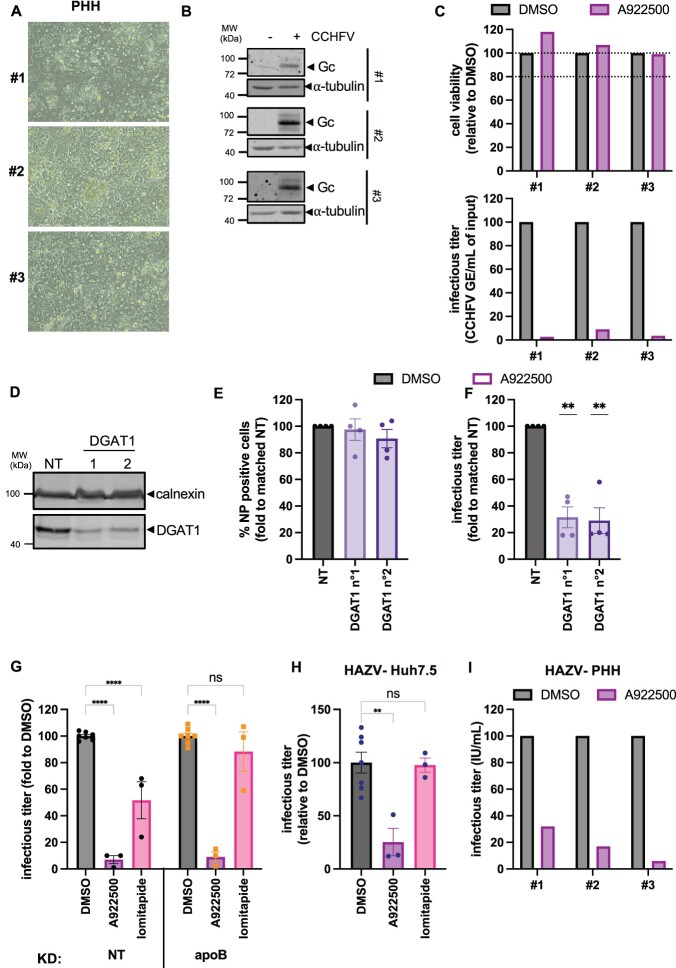
DGAT-1 is a proviral factor. (A) 3 batches of primary human hepatocytes (PHH) were infected with CCHFV. (B) Expression of intracellular CCHFV Gc was assessed at 24 h post-infection by Western Blot. (C) The batches of primary human hepatocytes (PHH) described in (A) were treated with 75 µM A922500 (batch #1 and #2) or 100 µM (batch #3), and cell viability was assessed at 24 h post-treatment. Dotted lines indicate reference thresholds (80%, 100%) and are included to facilitate interpretation of the data. PHH were infected with full-length CCHFV particles for 1 h before drug treatment with DMSO, 75 µM A922500 (batch #1 and #2) or 100 µM (batch #3). Supernatants were harvested at 24 h post-infection for evaluation of extracellular infectious titres on Huh-7.5 cells. Infectious titres of virus produced in cells treated with the inhibitor are presented after normalization to infectious titres of virus produced in cells treated with DMSO only. (D) Intracellular levels of DGAT-1 were assessed by Western blots of control Huh-7.5 cells (NT) or of Huh-7.5 cells transduced with two DGAT1-targeting shRNA-expressing lentiviral vectors. (E) Huh-7.5 cells described in (D) were used for the production of CCHFVmg particles. Percentage of CCHFV NP in transfected cells assessed by flow cytometry. (F) Infectious titres of CCHFVmg-GFP particles produced in Huh-7.5 cells, as described in (D), were assessed by flow cytometry. For (E) and (F), results are expressed as fold relative to NT. Results from 4 independent experiments. One-sample t-test. (G) Huh-7.5 cells were transduced with an apoB**-**targeting shRNA-expressing lentiviral vector. At 16 h post-transduction, cells were transfected with pCAGGS-NP, pCAGGS-V5-L, and pCAGGS-GPC. At 48 h post-transfection, cells were infected with CCHFVmg-GFP at MOI = 1. 3 h after infection, cells were treated with A922500 or lomitapide. 24 h after infection, supernatants were collected and used for titration in Huh-7.5 cells pre-transfected with pCAGGS-NP and pCAGGS-V5-L plasmids. Infectious viral titres from cells treated with the indicated inhibitors, normalized to the mean values obtained with DMSO for either NT or apoB KD. Results from 3 independent experiments. (H) Huh-7.5 cells were infected with HAZV particles for 1 h before drug treatment with DMSO, A922500, or lomitapide. Infectious viral titres from cells treated with the indicated inhibitors, normalized to the mean values obtained with DMSO. Results from 3 independent experiments. One-way ANOVA with Dunnett’s multiple comparison. (I) PHH from the three batches of (A) were infected with HAZV particles for 1 h before drug treatment with DMSO or A922500 as described in (C). At 24 h post-infection, supernatants were collected and used for titration in Huh-7.5 cells. Infectious viral titres from cells treated with the indicated inhibitors, normalized to the mean values obtained with DMSO. For (E–H), data are shown as means ± SEM, and each dot in the graphs corresponds to the value of an individual experiment.

First, we assessed the antiviral activity of A922500 in primary human hepatocytes (PHH), which are considered the most relevant ex vivo model to study interactions between pathogens and lipid metabolism. For that, we infected with WT CCHFV the PHH from 3 healthy donors (Figure 7A), which could efficiently replicate CCHFV as assessed by the expression of Gc in cell lysates (Figure 7B), before A922500 treatment at a non-toxic dose (Figure 7C) and assessment of the production of infectious particles. Depending on the donor, we could observe an up to 36-fold reduction (mean: 25-fold) in the production of infectious CCHFV particles (Figure 7C), confirming that A922500 is a potent antiviral molecule.

Second, to confirm that as a factor targeted by A922500, DGAT-1 is involved in the modulation of CCHFV infectivity, we induced its down-regulation in CCHFVmg-GFP producer cells using a shRNA-expressing lentiviral vector (Figure 7D). We found that, compared with control cells, DGAT-1 KD reduced the production of infectious particles (Figure 7E–F), in agreement with the results of DGAT-1 inhibition.

Third, we thought that the particularly potent antiviral effect of A922500, in addition to inhibiting lipoprotein synthesis, might be due to a more general impact on the modulation of triglyceride metabolism. Thus, to address this possibility, we inhibited the lipoprotein synthesis pathway through apoB down-regulation, and produced CCHFVmg-GFP particles in apoB KD cells that were treated with either DGAT-1 (A922500) or MTP (lomitapide) inhibitors. We found that while lomitapide modestly inhibited production of CCHFV particles from apoB KD cells, likely reflecting further inhibition of lipoprotein synthesis, A922500 induced a strong antiviral effect, suggesting a more general role of triglyceride metabolism in CCHFV infection (Figure 7G).

Finally, we thought that DGAT-1 inhibition may also have an antiviral effect on HAZV, an orthonairovirus that does not depend on the lipoprotein pathway for its production (Figure 2) and entry [7]. We found that while MTP inhibition did not prevent HAZV production (Figure 7H), treatment of HAZV producer cells with A922500 reduced by ca. 4-fold the formation of HAZV infectious particles both from Huh-7.5 cells (Figure 7H) and PHH (Figure 7I).

Altogether, these results indicated that inhibition of DGAT-1 could prevent, at different levels, the formation of CCHFV particles in a mechanism not only linked to lipoprotein metabolism.

## Discussion

### Heterogeneous population of CCHFV particles

Our results show that CCHFV is produced by hepatocytes as heterogeneous populations of viral particles, with two main populations, one of high density and one of low density, which is sensitive to apoB and apoE antibodies (Figure 3). We demonstrate that the low-density subpopulation of CCHFV particles incorporates apoB and that apoB KD blocks their production and egress, indicating their association with lipoprotein components (Figure 2). Furthermore, we show that the density of viral particles is influenced by serum components of the extracellular environment (Figure 4), suggesting that CCHFV particles can further be modified outside the producer cells, after production, in a manner reminiscent of hepatitis C virus [19,20]. Altogether, these results indicate for the first time that CCHFV particles could be subjected, in a proviral manner, to association with lipoprotein components, during their assembly and secretion steps, and, though not exclusively, to post-egress exchanges of components with lipoproteins contained in the serum, in a manner reminiscent of the maturation processes of lipoproteins themselves. We note that HAZV does not use the lipoprotein pathway to produce its viral particles, as shown by apoB neutralization, apoB KD, and MTP inhibition studies, in line with our previous results that HAZV does not use LDL-R for cell entry [7].

Despite their heterogeneity, all sub-populations of CCHFV particles appeared to be dependent on LDL-R for cell entry (Figure 1). Interestingly, previous studies by us [7] and others [8] showed that binding to LDL-R is mediated by both apoE associated with viral particles and by the CCHFV Gc surface glycoprotein, and that the presence of apoE enhances viral entry [7]. Our report not only sheds further light on this unexpected link with apolipoproteins but also reveals the intricacy between CCHFV assembly and secretion with the lipoprotein production pathway. Indeed, in contrast to apoE, an exchangeable apolipoprotein, apoB is a structural, non-exchangeable component of VLDLs and LDLs, though, like apoE, it also acts as a ligand of their receptors. Accordingly, as we show that antibodies against apoB or apoE preferentially blocked the infectivity of low-density CCHFV particles, this implies that the latter viral particles may preferentially bind LDL-R via apoB or apoE, while the high-density population may preferentially bind LDL-R via Gc.

These results may underscore an original viral strategy in which, depending on the producer cell type and/or host environment, CCHFV may co-opt the appropriate factors either in producer cells or in the extracellular milieu to maximize infectivity in a manner dependent on host conditions. In the case of human hepatocytes, which can readily produce infectious viral particles both as established cell lines [21] and as primary cells (Figure 7), the lipoprotein metabolic environment they provide seems particularly suitable for CCHFV. Further studies will be needed to elucidate the mechanism by which CCHFV could co-opt this pathway and the potential proximity of the viral assembly site to the intracellular localization of apoB (Figure 2J–K) in order to promote its production.

### Lipoprotein metabolism and alternative CCHFV host species

Lipid transport by lipoproteins is highly conserved among mammals, although lipoprotein lipidomes can differ in some species, including bovines, compared with humans [22]. For example, suggesting this relative conservation, bovine apoB shares about 74% of identity with human apoB. In that respect, as cattle and many other large mammals act as hosts for CCHFV and determinants of transmission to humans through, e.g. blood exposure [23,24], it would be interesting to study whether CCHFV particles have developed a specific mechanism for associating with human lipoprotein components only or, alternatively, whether this property is shared by all mammalian hosts and, if so, whether such association with their lipoproteins can promote infection in humans.

CCHFV can also be transmitted from mammals to humans via tick bites, which is considered to be its primary mode of transmission [3]. While the transport of lipids via lipoproteins is well described for mammals, their transport pathway has been poorly investigated in ticks. Yet, several reports have identified and characterized lipid carrier proteins in tick haemolymph, such as the haemolymph lipoprotein (HeLp) [25–29], similar to those described in insects. Like for mammals, such proteins are found associated with different types of lipids, including neutral lipids as well as free cholesterol or phospholipids. Thus, it would be interesting to determine if CCHFV particles can associate with such carrier proteins in its vector species and if this may promote infection in humans or, alternatively, if CCHFV is only able to associate with the human apoB, which boosts its infectivity and perhaps pathogenicity upon its transmission to this host and to its liver.

### CCHFV and the liver environment: an organ that boosts virus production and infectivity?

The liver, and in particular hepatocytes, is targeted by several viral pathogens that can impair its basic functions by inducing liver inflammation, liver degeneration, hepatocellular carcinoma, and, ultimately, liver failure. CCHFV infection induces massive liver injury, and hepatocytes are known to be the main targets of infection [4,30–32]. In addition, hepatocytes are also major sites of production of lipoproteins, with secretion of apoB- and apoE-containing VLDLs. Our results highlight, for the first time, an interplay between CCHFV production and the lipoprotein synthesis pathway and reveal the proviral roles of apoB, MTP, DGAT-1, and ACAT-1/2, which are crucial host determinants of VLDL production in the liver. How these host factors intervene to promote the production of CCHFV and/or its association with lipoprotein components is not known. One possibility is that CCHFV interacts early in its production steps with the VLDL assembly machinery (Figure 2J–K), e.g. at the stage of envelopment of viral particles, to recruit both apoB and apoE and perhaps other lipoprotein components. Alternatively, and in a non-exclusive manner, CCHFV may exploit some of the maturation steps of lipoproteins, once they are secreted out of the hepatocytes, to recruit these factors (Figure 4E–F). Yet, while apoE, an exchangeable apolipoprotein, can readily be transferred between different types of lipoproteins, which would explain how it can be recruited by CCHFV from the extracellular environment after its production, it is more challenging to figure out how apoB could be associated with the CCHFV surface, as this non-exchangeable apolipoprotein cannot be transferred [11]. Finally, our data underscore how the assembly of CCHFV infectious particles is more generally connected to lipid metabolism and, particularly, triglyceride synthesis, which is essential not only for lipoprotein production but also for many other cellular pathways. While we did not dissect the mechanisms involved, we note that triglyceride metabolism and, more specifically, DGAT-1 are crucial for promoting the infectivity of CCHFV (Figure 7) and of several other viral pathogens that may rely on lipid homeostasis or, alternatively, DGAT-1 proviral functions to ensure the production of infectious particles [33–36].

### The lipoprotein and triglyceride metabolic pathways: targets for developing a first-line antiviral strategy?

Our data led us to investigate whether targeting lipoprotein metabolism and, more generally, lipid metabolism could be a potential antiviral strategy, which could be highly beneficial in the case of CCHFV. First, host-targeted interventions could offer an effective approach against this emerging pathogen in the absence of approved direct-acting antivirals. Second, the metabolism of both lipoproteins and triglycerides has been widely studied. Indeed, since the disruption of lipoprotein metabolism is the cause of many non-infectious pathologies, such as hypercholesterolaemia, several molecules have been specifically developed, clinically validated, and marketed. In the same manner, molecules targeting DGAT-1 have been highly investigated to prevent non-infectious pathologies, such as obesity and coronary heart diseases. Even if some molecules are not currently used to treat these chronic diseases due to their side effects in long-term treatments, it is important to note that in the case of CCHFV infection, which is acute, their use would be very brief, hence limiting the induction of side effects. It is interesting to note that we discovered an antiviral effect of lomitapide (Figure 5C), an inhibitor of MTP, currently used in the treatment of familial hypercholesterolaemia [37]. Importantly, our results also reveal a potent antiviral effect of a small molecule inhibitor targeting DGAT-1, highlighting the translational potential of compounds already evaluated in phase I clinical trials [38–42].

Given these promising results, we extended our tests on molecules targeting nuclear liver factors, such as FXR, HNF4α, or PPARα, as these proteins are key regulators of liver metabolism, including lipoprotein production [43–47]. We observed a strong antiviral effect of the lipid-modulating drug gemfibrozil and the natural bile acid CDCA (Figure 6B–C). Gemfibrozil is clinically used to treat hypertriglyceridaemia, whereas CDCA is used to treat cerebrotendinous xanthomatosis, a rare autosomal recessive bile acid disorder. Interestingly, CDCA is a natural ligand of FXR; yet, we observed only a moderate effect of GW4064, a synthetic ligand of FXR (Figure 6B). This could be explained by the role of CDCA in the regulation of lipoprotein metabolism independently of FXR activation, as it was proposed previously [45].

In conclusion, our study reveals an opportunistic interaction of CCHFV production with lipoprotein and triglyceride metabolism . On this basis, we have demonstrated that small molecule inhibitors targeting lipoproteins, lipids, and, more generally, liver metabolism prevent the formation of infectious particles, which we validated ex vivo in primary human hepatocytes. It is clear that further work will be needed to characterize the mode of action of these lead inhibitors, as well as to validate alternative molecules targeting liver metabolic pathways or to improve their potency by, e.g. appropriate chemical modifications. Although our study did not confirm the efficacy of these molecules in vivo, in small animal models that would need to be housed in our BSL-4 laboratory, it is also important to note that, as hepatic metabolism differs between mice and humans, in vivo validation of these molecules will require the development of an infectious mouse model with a humanized liver, which has not yet been developed for CCHFV.

## Supplementary Material

Supplementary informations v2.pdf
