# Supplementary material for: The Crimean-Congo haemorrhagic fever virus hijacks the liver lipid metabolic pathway for virion production
Source: Emerg Microbes Infect. 2026 Mar 30;15(1):2645855. doi: 10.1080/22221751.2026.2645855 (PMC13037226; doi:10.1080/22221751.2026.2645855)
Supplement: Supplementary informations v2.pdf [file TEMI_A_2645855_SM5176.pdf]

Supplementary Table 1. Antibodies and molecules used in this study

Reagents	Concentration	Company (catalog number)
**Antibodies**		
LDL-R (goat IgG)	blocking assay: 4 ug/mL	R&D systems (AF2148)
apoE (goat serum)	neutralization assay: 1/100	Sigma-Aldrich (AB947)
apoB (goat serum)	neutralization assay: 1/100 – IP 10 uL – WB: 1/1000 – IF: 1/100	Sigma-Aldrich (AB742)
Goat serum	neutralization assay: 1/100 – IP 10 uL	Viromed (79S094)
Goat IgG	blocking assay: 4 ug/mL	ThermosFisher (02- 363 6202)
CCHFV Gc 11E7 (mouse)	WB: 1/1000 – IF: 1/200	BEI Resources (NR-40277)
CCHFV NP 9D5 (mouse)	FACS: 1/500	BEI Resources (NR-40277)
α-tubulin	WB: 1/10,000	Signalway Antibody (49624)
Calnexin (rabbit)	WB: 1/1,000	Enzo Life sciences (ADI-SPA-865-F)
DGAT1 (rabbit)	WB: 1/1,000	Abcam (ab178711)
**Molecules**		
lomitapide	100 nM	Sigma-Aldrich (SML1385-5MG)
A922500	10 uM	Sigma-Aldrich (A1737-1MG)
mipomersen	5 uM	MedChemExpress (HY-108764)
PF-06424439	10 uM	Cayman Chemical (17680)
Avasimibe	0.4 uM	Bio-Techne (6505)
GW4064	1 uM	MedChemExpress (HY-50108)
CDCA	200 uM	MedChemExpress (HY-76847)
Gemfibrozil	40 uM	Sigma-Aldrich (G9518-5G)
BI6015	10 uM	MedChemExpress (HY-108469)
ribavirin	100ug/mL	Sigma-Aldrich (PCT1133)
**Primers**	**Sequences**	
CCHFV	5′-CCCCACACCCCAAGATAATA-3′	5′-ACTACTCTGCATTCTCCTCA-3′
HAZV	5′-CAAGGCAAGCATTGCACAAC-3′	5′-GCTTTCTCTCACCCCTTTTAGGA-3′
GAPDH	5′-AGGTGAAGGTCGGAGTCAACG-3	5′-TGGAAGATGGTGATGGGATTTC-3′
XEF	5′-CGACGTTGTCACCGGGCACG-3′	5′-ACCAGGCATGGTGGTTACCTTTGC-3′

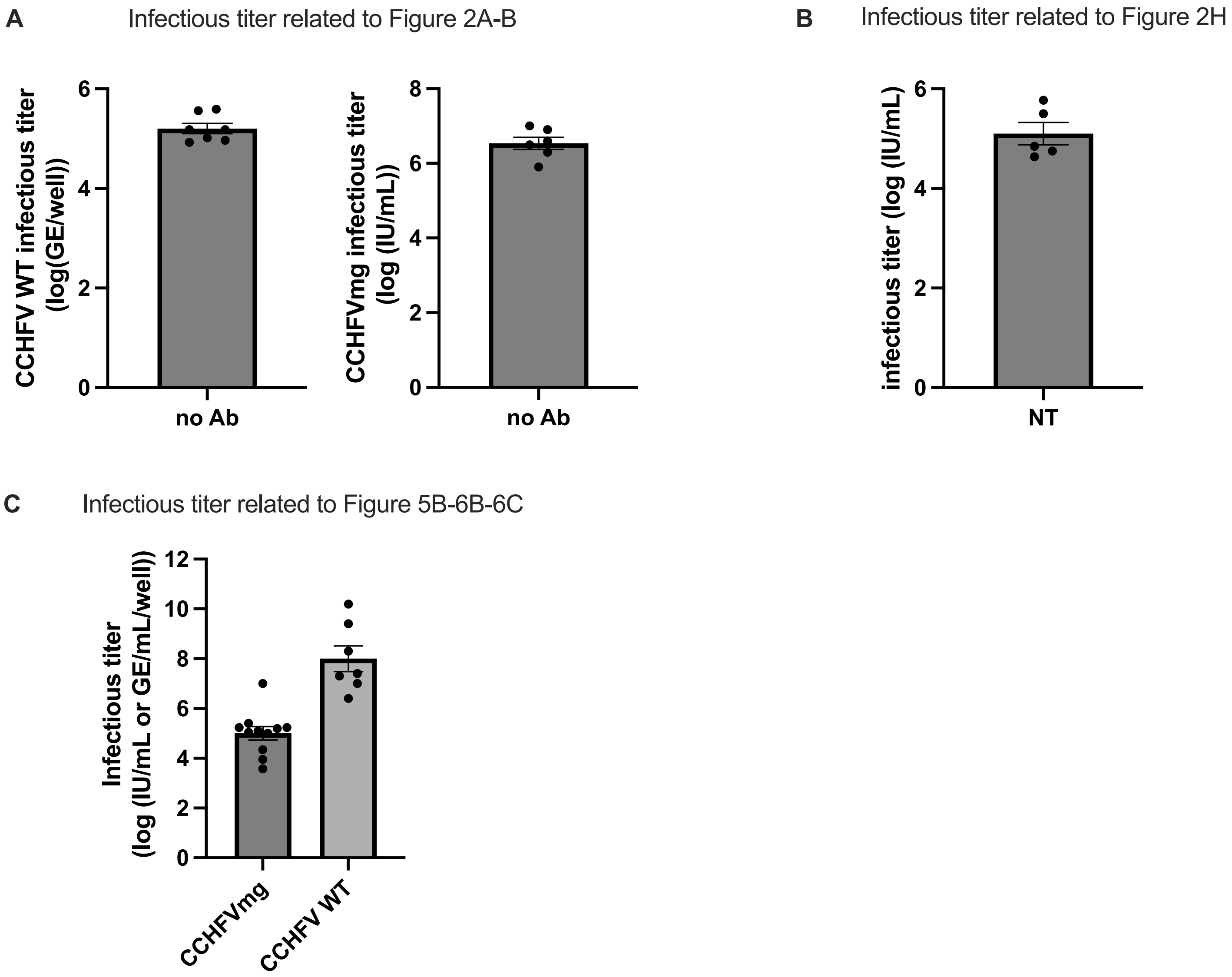

Supplemental Figure 1. Raw infectious titers.

(A) Infectious titers obtained for experiments depicted in Figure 2A-B. **(B)** Infectious titers obtained for experiments depicted in Figure 2H. (**C)** Infectious titers obtained for experiments depicted in Figures 5B-6B-6C.

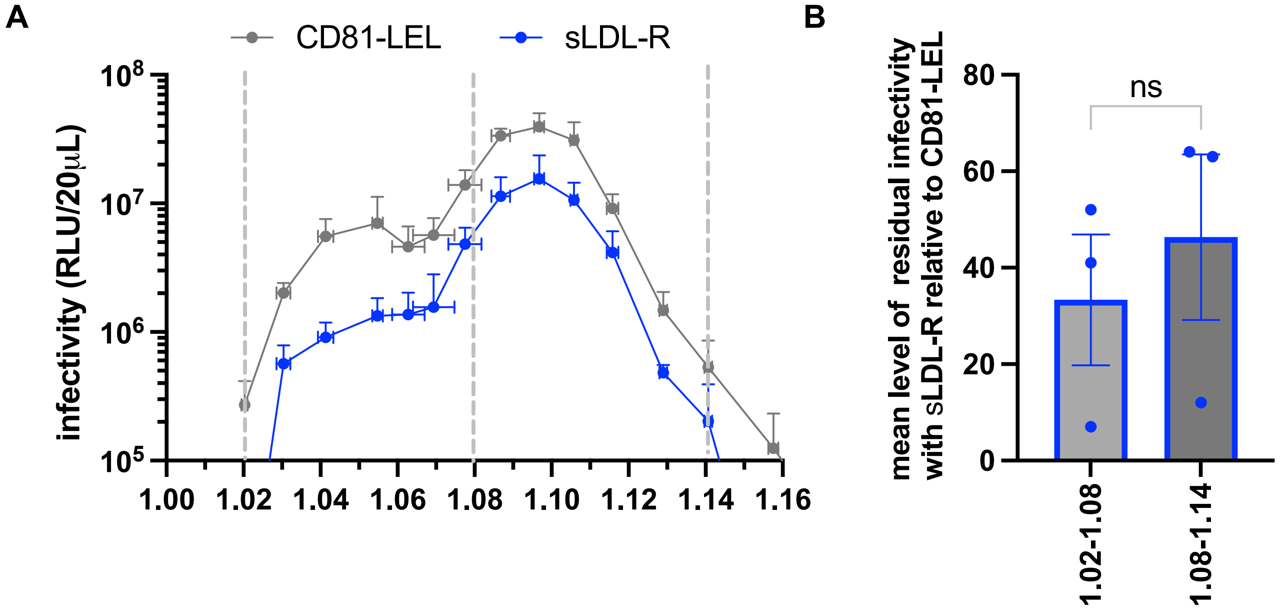

Supplemental Figure 2. Soluble LDL-R can neutralize CCHFV particles of both high- and low-densities.

(A) Fractions from gradients as described in Figure 1A were incubated for 1 h at room temperature with soluble LDL-R (sLDL-R) (blue) or with control protein CD81-LEL (grey) before infection. Cells were harvested at 24 h post-infection and infectivity was determined by measurement of nanoluc signals. The results display the means of infectivity in each fraction from different gradients. Paired ratio t-test. **(B)** Level of residual infectivity with sLDL-R relative to control protein from (A) was evaluated for each fraction and the mean level was determined for two categories: low-density fractions (1.02-1.08) and high-density fractions (1.08-1.14). Paired ratio t-test.

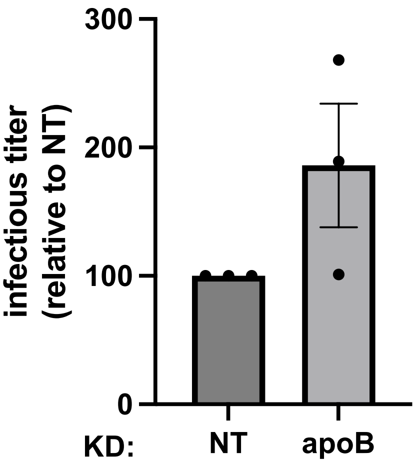

Supplemental Figure 3. apoB KD did not impair HAZV production.

(A) Control Huh-7.5 cells or Huh-7.5 cells transduced with an apoB-targeting shRNA-expressing lentiviral vector were used for production of HAZV. Infectious titer of HAZV produced in these cells as assessed by flow cytometry.

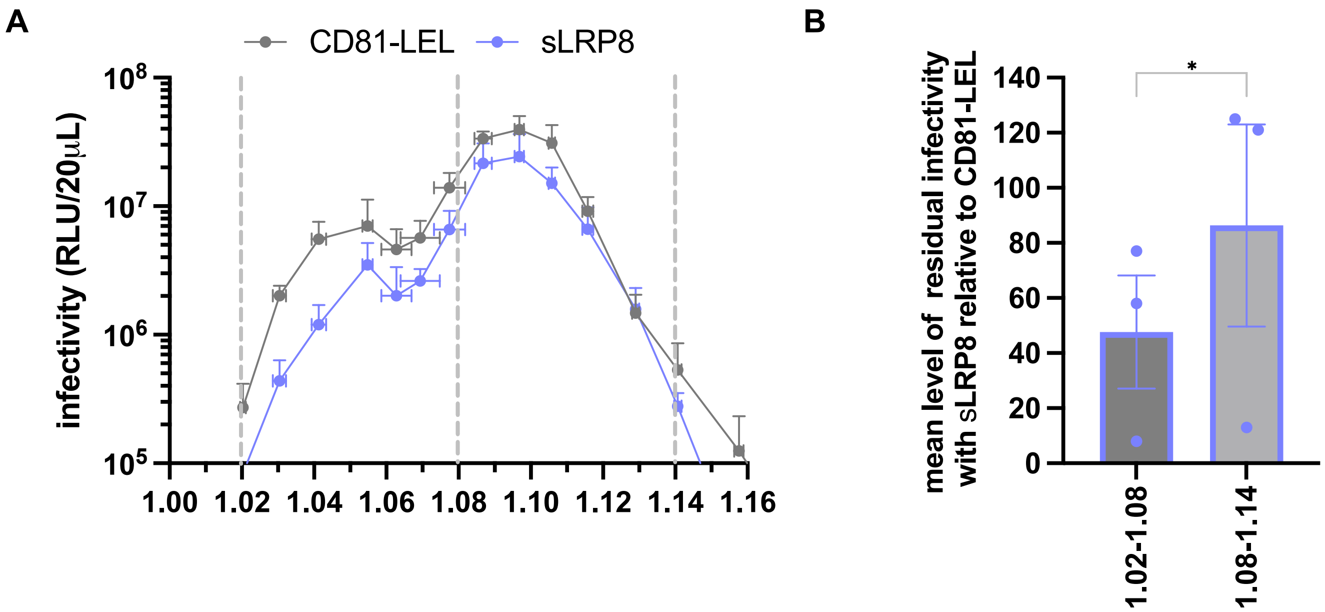

Supplemental Figure 4. Soluble LRP8 can neutralize CCHFV particles of low-densities.

(A) Fractions from gradients as described in Figure 1A were incubated for 1 h at room temperature with soluble LRP8 (sLRP8) (violet) or with control protein CD81-LEL (grey) before infection. Cells were harvested at 24 h post-infection and infectivity was determined by measurement of nanoluc signals. The results display the means of infectivity in each fraction from different gradients. Paired ratio t-test. **(B)** Level of residual infectivity with sLRP8 relative to control protein from (A) was evaluated for each fraction and the mean level was determined for two categories: low-density fractions (1.02-1.08) and high-density fractions (1.08-1.14). Paired ratio t-test.

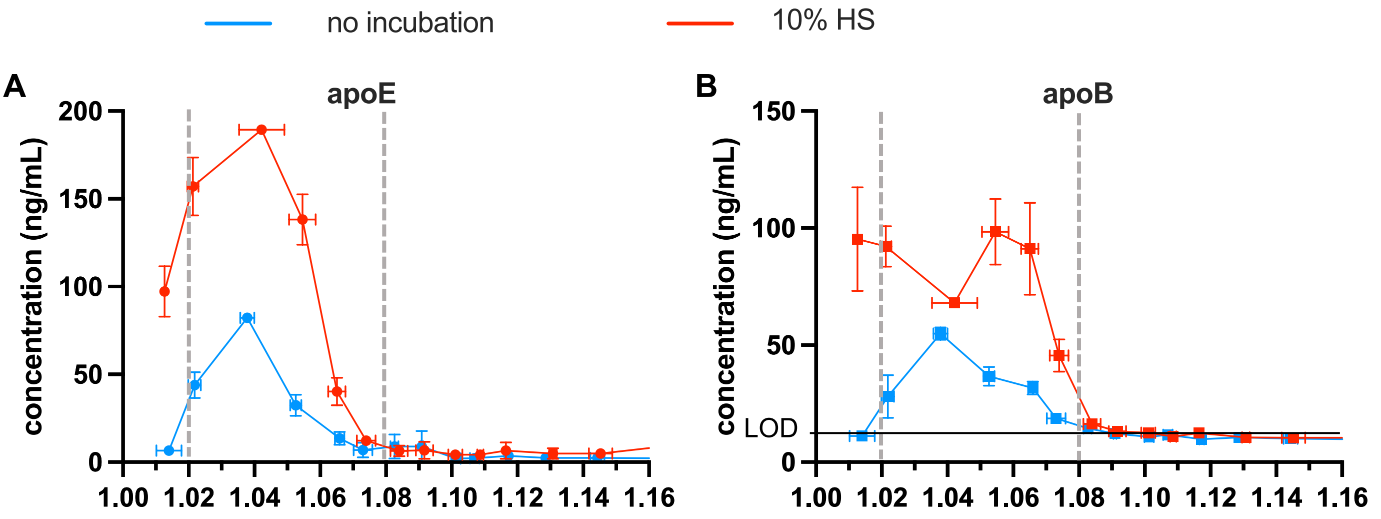

Supplemental Figure 5. apoE and apoB are found in low density fractions.
Fractions from gradients as described in Figure 4E were analyzed for their level of apoE **(A)** and apoB **(B)** by ELISA. Blue curves correspond to gradients from particles produced without serum, while red curves to gradients from particles produced without serum and incubated with 10% HS.
